# The Role of Clock Genes in Maintaining Circadian Rhythm and Rheumatoid Arthritis Pathophysiology

**DOI:** 10.7759/cureus.39104

**Published:** 2023-05-16

**Authors:** Arathi Kulkarni, Michelle Demory-Beckler, Marc M Kesselman

**Affiliations:** 1 Internal Medicine, Nova Southeastern University Dr. Kiran C. Patel College of Osteopathic Medicine, Davie, USA; 2 Division of Immunology, Nova Southeastern University Dr. Kiran C. Patel College of Allopathic Medicine, Davie, USA; 3 Rheumatology, Nova Southeastern University Dr. Kiran C. Patel College of Osteopathic Medicine, Davie, USA

**Keywords:** cortisol, sleep, circadian rhythm, clock genes, rheumatoid arthritis

## Abstract

Rheumatoid arthritis (RA) is a chronic, progressive autoimmune condition that affects up to 1% of the world population and symmetrically affects the joints leading to joint stiffness and decreased mobility. RA patients present with increased pain and chronic inflammation within their joint spaces, which researchers have linked to poorer sleep patterns, including difficulty falling asleep and non-restorative sleep. As such, identifying mediators of poor sleep quality among RA patients may improve their long-term quality of life. More recently, researchers identified an association between chronic inflammation in RA patients and their circadian rhythm. Altered circadian rhythms negatively impact the hypothalamic-pituitary-adrenal (HPA) axis and lead to altered cortisol release. Cortisol has shown to have a strong anti-inflammatory effect; when dysregulated, it may lead to increased pain experienced in RA patients. This literature review aims to provide insight into how chronic inflammation tied to RA pathophysiology may affect clock genes that are involved in maintaining the circadian rhythm. Specifically, this review focused on four common clock genes found dysregulated in RA patients: circadian locomotor output cycles kaput (*CLOCK*)*, *brain and muscle ARNT like-1* *(*BMAL1*)*, *period* *(*PER*)*, *and cryptochrome (*CRY*). Of the four clock genes discussed in this review, *BMAL1 *and *PER *are the most well-studied of the affected genes. Further knowledge surrounding clock genes and their dysregulated expression in RA may help guide therapy decisions for RA patients. Traditionally, disease-modifying antirheumatic drugs (DMARDs) have been used as first-line therapy for RA patients. Meanwhile, chronotherapy, optimizing drug release in a timed manner, has shown positive results in RA patients as well. Because of the association of altered circadian rhythms with increased symptom severity in RA patients, it seems highly plausible that DMARD therapy with chronotherapy may be an ideal therapeutic regimen for RA.

## Introduction and background

**Figure 1:**
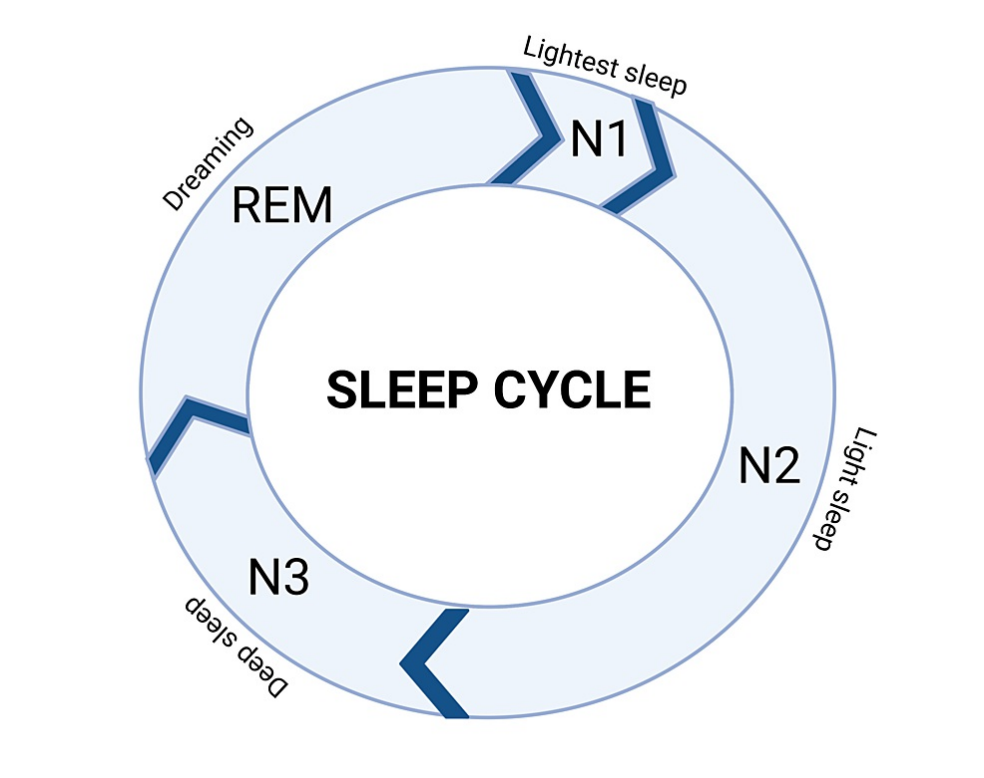
Sleep cycle across a 24-hour time period Original image created using BioRender.com

Rheumatoid arthritis (RA) is a chronic inflammatory condition that affects the synovial tissue of joints, cartilage, and extra-articular tissue, with presenting symptomatology including synovitis and pain [1]. Genetic and environmental factors both play a role in RA pathogenesis. Clinically, patients may present with symmetric wrist, proximal interphalangeal joint, and metacarpophalangeal joint swelling. Lab findings often include the presence of anti-citrullinated protein antibodies (ACPA) and rheumatoid factor (RF). Advances in the understanding of disease-modifying antirheumatic drugs (DMARDs) including conventional synthetic DMARDs, biologic DMARDs, and targeted synthetic DMARDs and other modes of treatment have helped many patients reach clinical remission [1,2]. However, RA patients still have a lower quality of life (QoL) when compared to their healthy counterparts due to comorbidities such as interstitial lung disease, cardiovascular disease, cervical myelopathy, and joint ankylosis, which contributes to increased mortality, significant disability, and lower QoL scores of RA patients [1,3-5]. In addition to these co-morbidities, the chronic inflammation associated with RA appears to be linked to alterations in RA patients’ sleep patterns [6]. The sleep cycle is separated into two primary phases: rapid eye movement (REM) and non-REM sleep [7]. Non-REM sleep is further divided into three different stages: N1, N2, and N3. Figure 1 outlines the stages of sleep. Over the course of the night, the body cycles through the phases and stages of sleep about four to six times, which ultimately leads to a refreshed night’s sleep [7]. 

Approximately 54-70% of RA patients report sleep issues [6], including poor quality of sleep, difficulty falling asleep, non-restorative sleep, sleep awakening during the night, wakefulness, and increased daytime sleepiness [6]. Sleep issues in RA patients appear to have multiple causes, including increased pain felt from the disease process [8]. Patients with chronic pain tend to experience less deep sleep and are 18 times more likely to experience clinically diagnosed insomnia [8,9]. In addition, chronically experiencing poor sleep can have compounding effects on an RA patient’s pain levels [9]. Long-term poor sleep quality can predispose patients to increases in low-grade inflammation and may be associated with greater disease activity and pain [6,9,10]. Identifying additional factors that contribute to poor sleep quality in patients with RA may be significant in improving their health-related QoL (HRQoL), a numerical score that can be measured using the 36-Item Short Form Survey (SF 36) [11].

**Figure 2:**
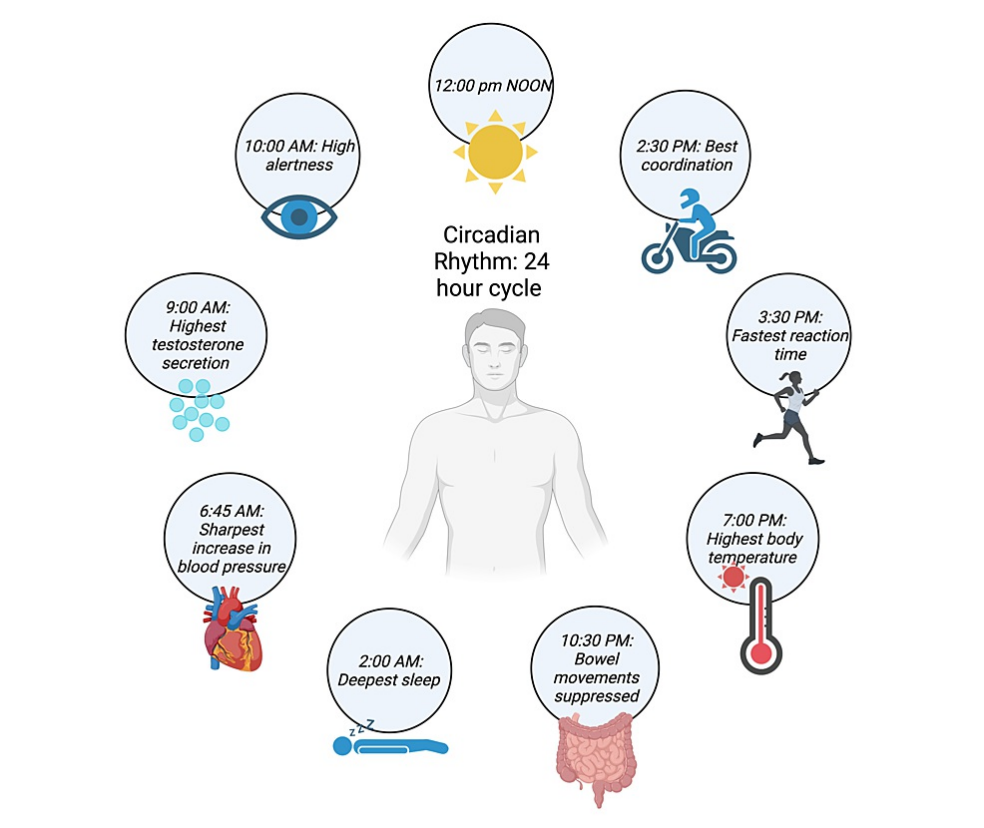
Circadian rhythm across a 24-hour time period Original image created using BioRender.com

**Figure 3:**
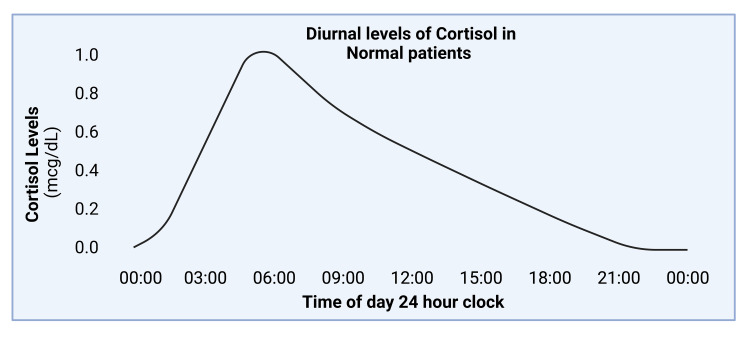
Average diurnal levels of cortisol across a 24-hour time period Original image created using BioRender.com

The circadian rhythm, the human body’s internal clock that guides homeostatic functions, plays a significant role in guiding sleep patterns (Figure 2). The circadian rhythm helps regulate our internal physiological functions such as the sleep-wake cycle, body temperature, cardiovascular system, hormones, and immunity [12]. The circadian cycle is based on a 24-hour day, with periods of light and dark (Figure 3). Light, in the form of sunlight or blue light, genetics, behavior, and inflammation all play a role in affecting the circadian rhythm [13]. Much research has shown disruption of circadian rhythms in chronic disease states. These include diabetes mellitus type 2, Alzheimer’s disease, and RA [13,14]. In addition, disruptions of the circadian cycle seem to have a variety of downstream effects. These include increased inflammation and pain, neurodegeneration, and metabolic changes [12,13].

The circadian clock is guided by the suprachiasmatic nucleus (SCN) in the hypothalamus of the brain. The SCN acts as the central pacemaker of the human body and guides the expression of clock genes in various tissues [15]. Clock genes play a critical role in regulating the circadian rhythm at the molecular level [15] and include circadian locomotor output cycles kaput (*CLOCK*), brain and muscle ARNT like-1 (*BMAL1*), period (*PER*), and cryptochrome (*CRY*) [15]. Specifically, *CLOCK *and *BMAL1 *appear to be positive regulators of clock processes, while *PER* and *CRY* appear to be negative regulators [15]. Alterations in these genes may affect the circadian rhythm including dysfunction in timed cortisol release. In RA, the circadian rhythm plays particular importance as its cycle helps control the release of hormones, including cortisol, an important mediator of the inflammation of RA [16]. Altered cortisol production in RA patients may lead to increased inflammation and, consequently, pain that disrupts their sleep.

Cortisol is a hormone that has one of the strongest anti-inflammatory effects in the hypothalamic-pituitary-adrenal (HPA) axis. The HPA axis guides neuroendocrine function by modulating the release of various hormones under the effects of stress [17]. The circadian rhythm helps mediate the timed release of cortisol (Figure 3); however, in RA patients, the circadian rhythm may be disturbed due to chronic inflammation and alterations in clock genes [15,18]. Neeck et al. found that diurnal cortisol levels during the day fluctuated in patients with RA based on the severity of their disease [16]. In mild to moderate cases, cortisol levels’ maxima and minima peaks were earlier in the day. In more severe cases, the circadian rhythm responsible for cortisol release was significantly reduced or lost [16]. Downregulation of cortisol in the evening and night may lead to the inflammatory effects of RA seen in the early morning including joint stiffness and pain. Morning joint stiffness and pain are also referred to as the gelling phenomenon.

Its second peak during the day may explain the inhibition of RA symptoms throughout the day [19]. In addition, patients with RA appear to have increased tumor necrosis factor alpha (TNF-α), which contributes to cortisol inhibition as well [20]. Moreover, Straub et al. found that anti-TNF-α antibodies interfere with the HPA axis and alter the amount of cortisol released in RA patients, which likely affects RA disease symptoms [19,20]. In addition, there was an associated increase in disease activity score in 28 joints (DAS28) tied with lower serum cortisol levels. When patients were followed longitudinally with prolonged anti-TNF-α therapy, patients with initially lower serum cortisol levels showed a marked increase in serum cortisol levels [19]. However, other studies have demonstrated that serum cortisol levels may not be altered in RA patients, but they may peak at a different time when compared to controls. Cutolo et al. evaluated 19 RA patients from Estonia and seven RA patients from Italy with similar disease duration and severity [21]. Serum cortisol levels from blood samples were monitored for two months at 8 PM, 10 PM, 12 AM, 2 AM, 4 AM, 6 AM, 8 AM, and 3 PM. No significant differences were found in serum cortisol levels between healthy controls and RA patients, but RA patients’ peak cortisol levels were approximately two hours after (8 AM) control patients (6 AM) [21]. Overall, these studies suggest that cortisol peak changes may correlate with increased TNF-α serum levels as well as worsening disease severity, which can be abrogated with anti-TNF-α therapy. However, future studies are needed that directly determine the effect of modified cortisol peaks on RA patients’ circadian rhythms, sleep quality, serum cytokine levels, and disease severity. As a first step, the goal of this systematic review is to more clearly elucidate the links between clock genes and RA.

## Review

Methods

**Figure 4:**
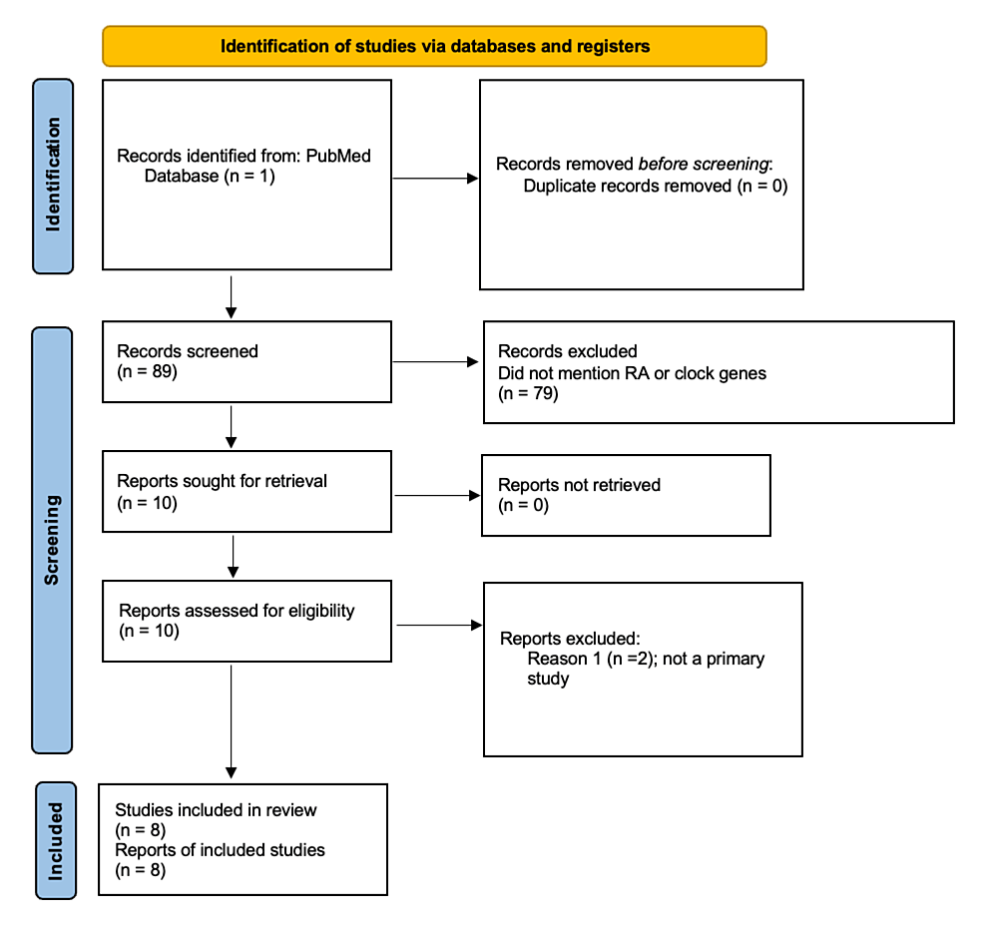
PRISMA chart of the eight articles chosen for review PRISMA, preferred reporting items for systematic reviews and meta-analyses

This review included eight primary studies gathered from PubMed’s online database. Overall, 89 articles were obtained, from which eight manuscripts were chosen for this review. The preferred reporting items for systematic reviews and meta-analyses (PRISMA) chart is shown below of the included studies (Figure 4). Key words used to identify relevant research articles included “clock genes and arthritis,” “clock genes and RA,” “clocks and RA,” “disruption of rhythms and RA,” and “Per2 and RA.”

Results

Animal Study: Circadian Rhythm Clock Genes and RA

Hashiramoto et al. evaluated the role of the circadian clock mouse genes *Cry1* and *Cry2* in circadian rhythm regulation in a mouse model and their effect on inflammatory arthritis [15]. To do this, inflammatory synovial cells and spleen cells of mice with experimental inflammatory arthritis were studied. The results show that mice lacking the two core clock genes *Cry1 *and *Cry2 *had more aggressive inflammatory arthritis, with increased production of TNF-α in spleen cells [15]. Once treated with anti-TNF-α antibodies, *Cry1−/−Cry2−/−* mice progression of arthritis was halted, suggesting that the circadian rhythm and arthritis affect each other, and disruptions in the circadian rhythm mouse clock genes *Cry1* and *Cry2* may hasten the progression of inflammatory arthritis [15].

Human Studies: Circadian Rhythm Clock Genes and RA With Lipopolysaccharide (LPS) Association

Human studies have also shown that clock genes may be disrupted in RA patients and may affect their circadian rhythm. A 2017 published study looked at the association between the clock gene *PER2* in RA and osteoarthritis (OA) patients [22]. Researchers analyzed three RA and three OA synovial cells. These cells were subsequently exposed to LPS to induce inflammation. Total protein was extracted from three RA patients’ synovial cells, and PER2 protein expression was assayed through immunoblotting. Researchers found that RA patients’ synovial cells, when compared to controls, did not have a significant difference in PER2 protein expression, but, when exposed to LPS to induce inflammation, PER2 protein expression declined in a time-dependent manner. Their results suggested that the *PER2* gene may be a risk factor for RA, and expression of the PER2 protein may be affected by inflammation [22]. 

*Human Studies: Circadian Rhythm Clock Genes and RA* * *

In a 2015 published study, five blood samples from five RA (DAS28 ≥ 4.2) and five blood samples from non-RA healthy controls were evaluated [23]. Samples were collected every two hours for 24 hours and analyzed for clock gene expression in isolated CD14+ monocytes through quantitative RT-PCR. The results showed that clock genes, *PER2* and *PER3*, in CD14+ monocytes did not exhibit circadian expression amplitude in patients with RA when compared to controls. The reported data suggest that some immune cell populations in RA lose their normal circadian rhythms, whereas others establish new inflammatory circadian rhythms [23].

Human Studies: Circadian Rhythm Clock Genes and RA With TNF-α Association

A 2012 study examined clock genes in synovial fibroblasts of 17 RA and 17 OA patients to better understand the effects of the proinflammatory cytokines interleukin 1 (IL-1) and TNF-α on the rhythmicity of the circadian clock by analyzing mRNA expression [24]. The presence of BMAL1, CLOCK, PER1, and PER protein was determined using immunofluorescence followed by analysis with quantitative PCR of mRNA. Researchers found all four clock genes to be expressed, and supplemental IL-1 and TNF-α supplementation stimulated the average mRNA expression of RA fibroblasts. However, IL-1 and TNF-α delayed the first peak of BMAL1 mRNA level expression in RA synovial cells by three to six hours. Researchers concluded that proinflammatory cytokines may influence the oscillation of mRNA expression in molecular clocks, but the period of oscillation could not be studied because the mRNA signal rapidly died down after initiation [24].

A 2013 published study evaluated 13 different clock genes located on the synovial membrane of joints in RA patients [18]. Tissue samples were collected from 10 RA and OA patients in the morning during synovectomy or surgery for complete joint replacement. The protein expression of clock genes neuronal PAS domain-containing protein 2 (*NPAS2*) and aryl hydrocarbon receptor nuclear translocator-like 2 (*ARNTL2*/*BMAL2*) was analyzed. Upon supplemental TNF-α exposure, investigators found that two genes, *NPAS2* and *ARNTL2*, were the most affected in RA tissue that correlated with circadian clock impairment. In addition, they found that the expression of *BMAL1* gene varies the most in RA patients during the day. The findings suggest that the circadian rhythm is disturbed on the molecular level in RA patients and that pro-inflammatory mediators can influence these molecular underpinnings indicating that inflammation may play a role in circadian timekeeping [18].

A 2018 published study examined the mechanism underlying the TNF-α-induced overexpression of clock gene *BMAL1* in RA patients [25]. Synovial tissues were obtained during joint surgery from 12 different patients with RA. The results showed that TNF-α altered the transcription, but not the circadian oscillation, of *BMAL1*, by up-regulating the gene RAR‐related orphan receptor (*RORA*). The researchers proposed that therapy directed toward limiting the expression of *BMAL1* could be a novel treatment for RA [25].

On the other hand, a 2014 published study demonstrated the presence of the clock genes *BMAL1*, *CLOCK*, *PER1*, and *PER2 *and albumin D-box binding protein (DBP) in three RA and three OA patients after exposure to TNF-α [26]. Researchers examined the clock genes in the synovial tissues of these patients using immunohistochemistry. Expression of the genes was determined through real-time PCR after a two-hour shock of TNF-α. The results show that TNF-α may not modify clock gene expression in synovial fibroblast cells of RA patients as they found the core clock genes were expressed similarly in RA and OA patients. These results suggest that the circadian rhythm may be intact even during inflammatory conditions [26].

Human Studies: Circadian Rhythm Clock Genes and RA With DMARD Therapy

DMARD therapy has also been shown to affect the circadian rhythm of RA patients by altering the presence of clock genes. A 2020 published study looked at how clock genes are affected by DMARD therapy in RA patients [27]. Fifteen RA patients treated with DMARDs were studied. Blood samples were collected from the RA patients prior to treatment at the start of the study and again at the conclusion of the study. Total RNA was extracted from leukocytes to examine the expressions of the clock genes. The researchers evaluated the correlation of the clock gene expression with disease activity by studying the expressions of the circadian clock genes in leukocytes of patients prior to and after treatment with DMARDs. Expressions of *PER2*, *CRY1*, *CRY2*, *CLOCK*, and *RORA *in RA patients were lower as compared with healthy controls. In addition, the DAS28 erythrocyte sedimentation rate (ESR) was negatively correlated with the expressions of *PER2*. Researchers concluded that the expression of the clock genes, specifically *PER2*, E-4 binding protein 4 (*E4BP4*), and *RORA*, serves as useful biomarkers in predicting RA disease activity and the efficacy of DMARDs in RA patients. The investigators proposed that the expression of clock genes in leukocytes could be useful as novel biomarkers, predicting disease progression and therapeutic effectiveness for DMARDs in RA treatment [27].

Discussion

This literature review suggests that RA patients appear to have altered circadian rhythms and dysregulated expression of various clock genes, namely *BMAL1* and *PER2*. Altered circadian rhythms may disrupt the timely release of cortisol, which leads to prolonged inflammatory effects and disrupted sleep that can dampen the QoL of RA patients. Six of the seven human studies and one animal study showed this association of dysregulated clock genes in RA patients through dysfunctional or delayed expression. One of these studies even proposed that the clock gene *PER2* may not only be affected by the inflammatory conditions of RA, but it may also be involved in the pathogenesis of RA due to its depressed expression in RA synovial cells [22]. This decreased expression may alter the circadian rhythm in RA patients and worsen inflammation and symptoms [22]. However, this association was not homogeneous in this literature review. Becker et al. found that the inflammatory conditions in RA may not affect clock gene expression in RA synovial tissue as they found similar clock gene expression in RA and OA patients’ synovial tissue [26]. While further research needs to be done on understanding clock gene expression in RA patients, the findings in these studies demonstrate that there may be a relationship between inflammatory conditions of RA patients and dysregulated clock gene expression affecting circadian rhythmicity.

Limitations of the studies selected for review include human studies, in which patients were receiving RA treatment, which may have affected clock gene analysis. Analysis of newly diagnosed RA patients may form a baseline of how extensive clock gene dysregulated expression is. Another limitation of the seven chosen human studies is that researchers did not disclose at what age each RA patient was diagnosed with RA. Time may have played a role in how extensive clock gene expression may have been dysregulated in RA patients who were diagnosed at an earlier age versus at a later age. Patients who have been living with RA longer may have more impaired clock genes compared to patients who have been newly diagnosed. Studies done with patients separated based on their age group at the time of RA diagnosis may better elucidate the generalizability of results.

There have been proposed treatments for altered circadian rhythms in RA patients. Traditionally, the standard treatment for RA is DMARD therapy, but chronotherapy, which involves optimizing the timing of drug administration according to target receptor and gene expression to maximize therapeutic efficacy and minimize side effects, has shown efficacious results as well. Studies have shown that chronotherapy of prednisone and methotrexate, when used in adjunct with traditional DMARD therapy, may improve symptom progression in RA patients. A 2010 published clinical study showed that nighttime methotrexate chronotherapy given three times a week in the evening can improve early morning RA symptoms compared to the current standard dosing methods [28]. Timing methotrexate therapy in synchronization with circadian rhythms may lead to more effective treatment in RA patients [29]. Chronotherapy with prednisone has also yielded positive results. In a 2013 randomized clinical trial, researchers studied how the circadian administration of prednisone (CAPRA-2) affects RA symptoms and severity. They found that low-dose prednisone chronotherapy for RA added to existing DMARD treatment helped significantly reduce morning stiffness, severity of symptoms, and fatigue [30]. RA patients identified with altered clock gene expression may benefit from corresponding treatment with chronotherapy to better manage symptoms and dysregulated sleep. Future research can be focused on expanding clock gene analysis in a larger subset of patients with a focus on *BMAL1* and *PER* genes. Chronotherapy with methotrexate and prednisone may then be used to assess its effects of timing on RA patients with impaired clock genes.

## Conclusions

The circadian rhythm in RA patients appears to be altered on the molecular level of clock genes. Changes in the circadian rhythm may lead to poorer sleep and lower QoL in RA patients. Chronotherapy may be an effective adjunctive treatment option for RA patients to achieve better overall symptom control. Better symptom control can lead to decreased pain and more efficacious nightly sleep. Treatment directed toward the circadian rhythm variabilities in RA patients may further improve their long-term QoL by improving pain control and sleep patterns.
